# Supplementary figures and images for: Wbm0152, an outer membrane lipoprotein of the Wolbachia endosymbiont of Brugia malayi, inhibits yeast ESCRT complex activity
Source: PLoS Pathog. 2025 Dec 11;21(12):e1013383. doi: 10.1371/journal.ppat.1013383 (PMC12707686; doi:10.1371/journal.ppat.1013383)

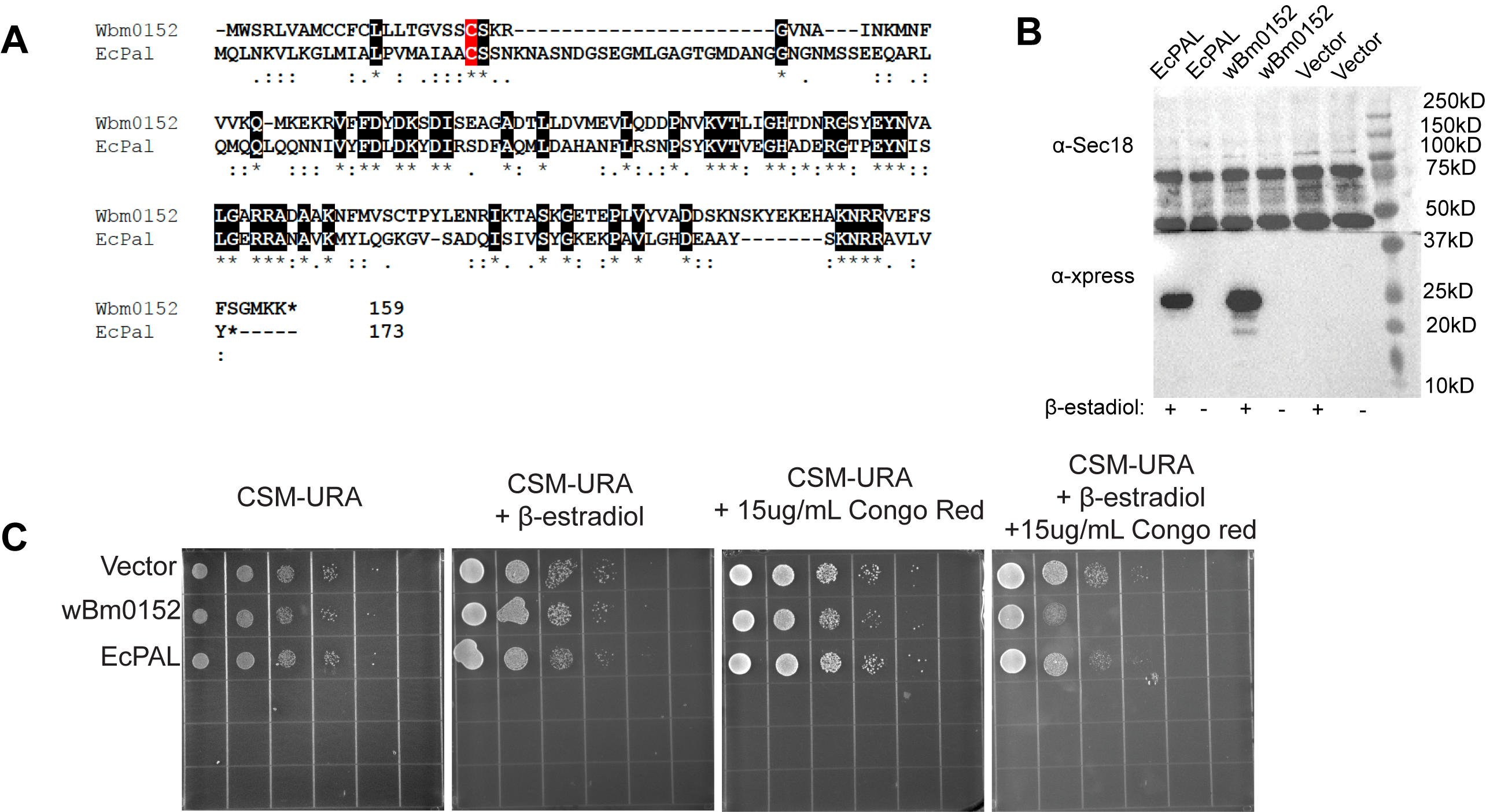

Supplement: S1 Fig — A) Sequence alignment of wBm0152 compared to E. coli homolog EcPAL in which highlighted residues (*) indicate identical amino acids, (:) indicate highly similar amino acids and (.) indicate similar residues between the two proteins. B) BY4742 strains containing either a beta estradiol inducible pYES-EcPAL, pYES-wBm0152 or empty vector control strain grown overnight shaking at 30° C, then subcultured into 5mL of CSM-URA media with and without 1 µM beta estradiol for four hours to induce expression. Whole cell lysates were collected and probed with anti-Xpress antibody to detect indicated protein and anti-Sec18 as a loading control. C) Strains were grown overnight at 30° C in CSM-URA media. Cultures were diluted to a final OD600 = 1.0, serially diluted 10-fold four times into sterile water, and 10 µL of each dilution was spotted to CSM media lacking uracil either lacking or containing 15 µg mL-1 Congo red. Plates were incubated at 30° C and imaged after 72h. (TIF) [file ppat.1013383.s001.tif]

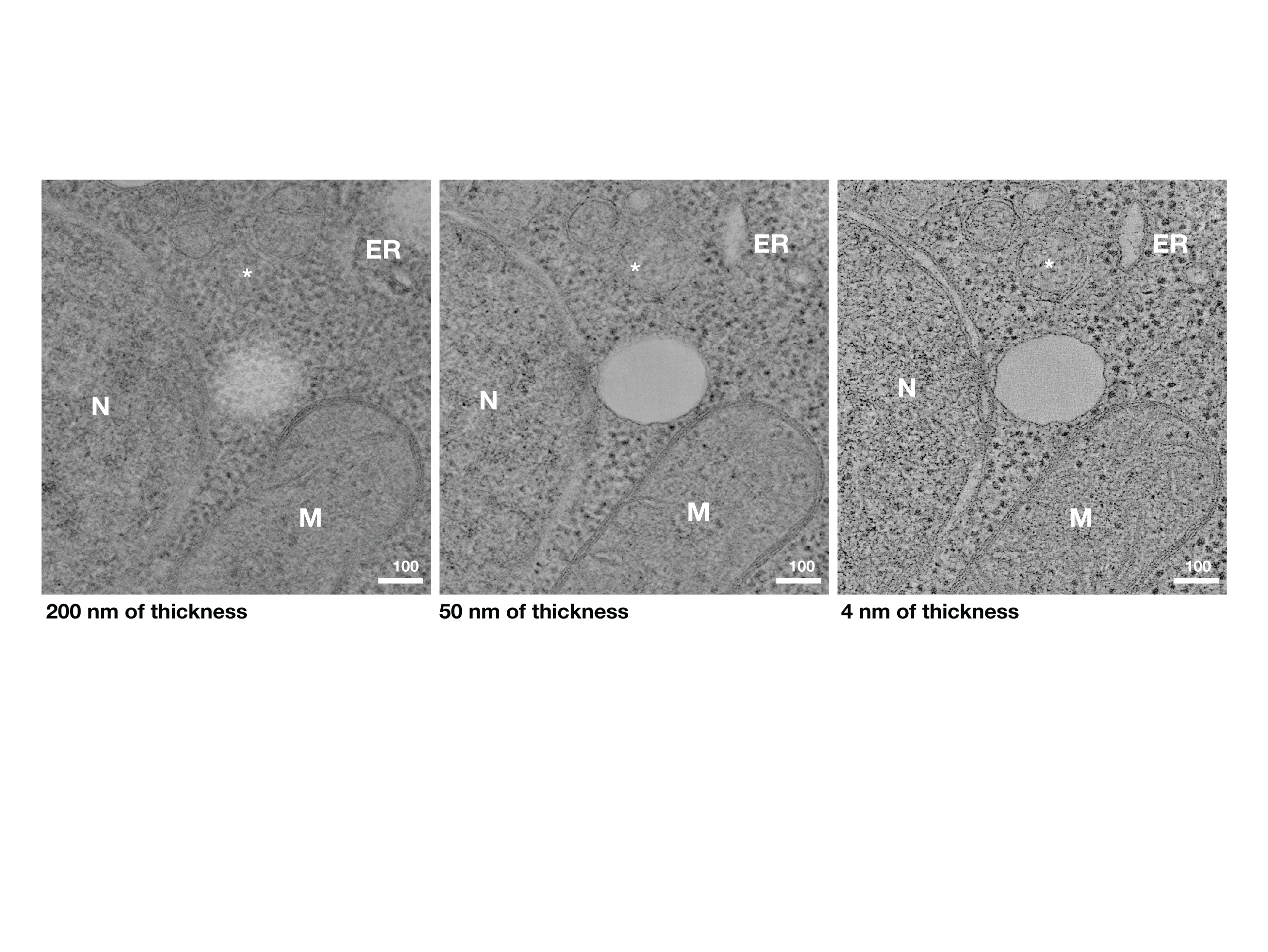

Supplement: S2 Fig — Cells from the wild type strain, SEY6210, were imaged at 200 nM (left), 50 nm (middle), and 4 nm (right), thicknesses of the tomographic volume, to visualize various membrane bound organelles. Organelles denoted as nucleus (N), mitochondria (M), endoplasmic reticulum (ER), and autophagosome (*). (TIF) [file ppat.1013383.s002.tif]

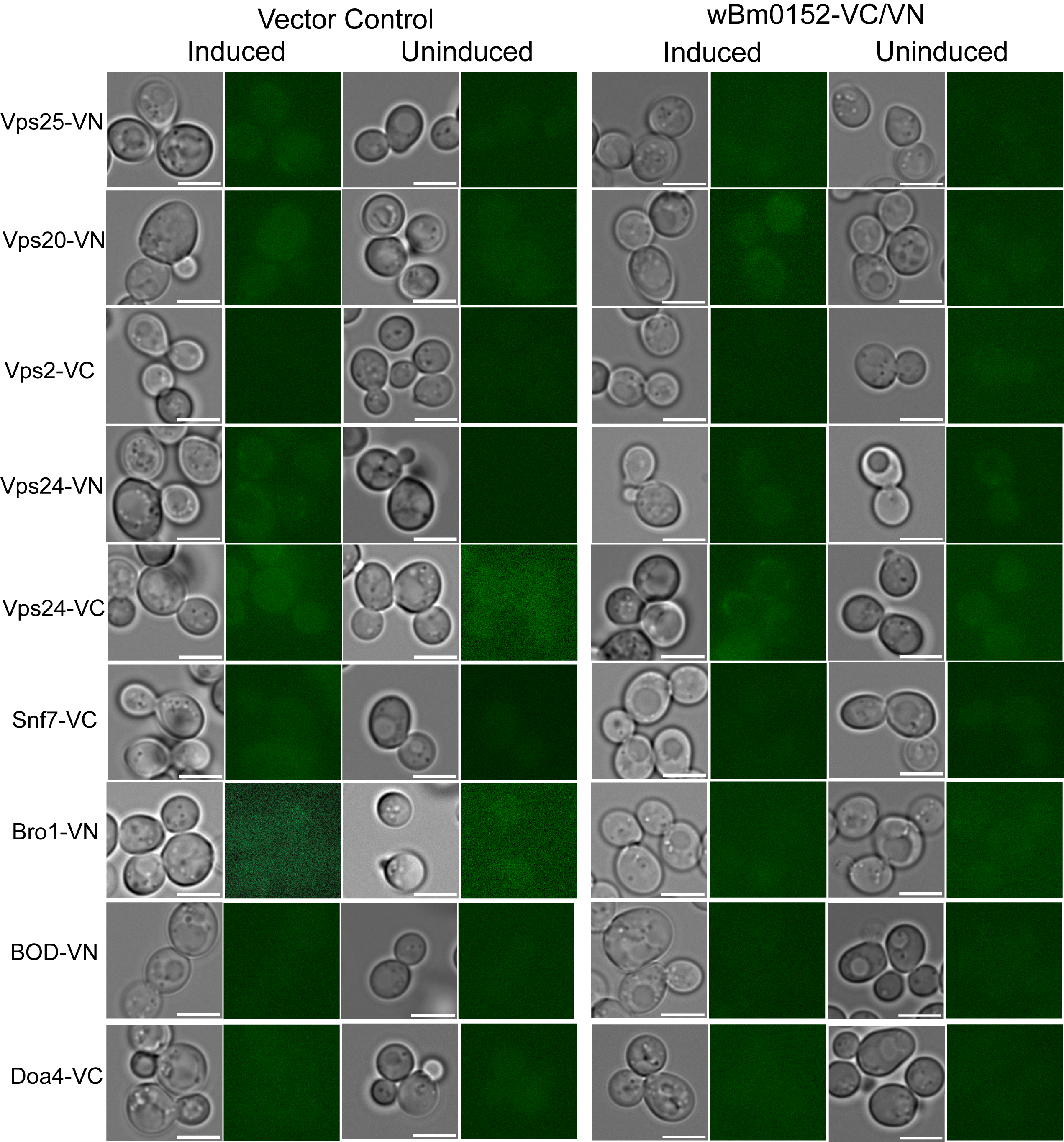

Supplement: S3 Fig — SEY6210 strains harboring either the N-terminus (VN) or C-terminus (VC) of a Venus-YFP molecule on the C-terminus of the indicated ESCRT subunit were transformed with the corresponding copper-inducible pYESCUP1-wBm0152-VN or pYESCUP1-wBm0152-VC plasmid. Strains were grown in CSM media lacking uracil for 18h at 30° C with shaking, diluted 1:10 into fresh selective media lacking or supplemented with 0.5 mM CuSO4, and outgrown for 6 hours before imaging. Bar = 5 µ; images are representative of three separate experiments. (TIF) [file ppat.1013383.s005.tif]
