# Supplementary material for: Wbm0152, an outer membrane lipoprotein of the Wolbachia endosymbiont of Brugia malayi, inhibits yeast ESCRT complex activity
Source: PLoS Pathog. 2025 Dec 11;21(12):e1013383. doi: 10.1371/journal.ppat.1013383 (PMC12707686; doi:10.1371/journal.ppat.1013383)
Supplement: S1 Table — (DOCX) [file ppat.1013383.s006.docx]

**S1 Table. Primer and nucleotide sequences used in this study^a^**

| **Primers** |  |  |
| --- | --- | --- |
| YTDH F | 5’-TACAGGGCGCGTGGGGATGATCCACTAGTATCATT  ATCAATACTCGCCATTTCAAAG | pYES TDH3 promoter conversion |
| YTDH R | 5’-ATGATGATGATGATGATGAGAACCCCCCATGGTAA  GCTTAATATTCCCTATAGTGAGTC |  |
| CUP  F | 5’-TCTGTTAGAAACCTTGACTATGGG | pYES CUP1 promoter conversion |
| CUP  R | 5’-GTTTCTCGGTCTAAGAGCTTATACG |  |
| YCUP  F | 5’- TACAGGGCGCGTGGGGATGATCCACTAGTACCGAT  CCCATTACCGACATTTG |  |
| YCUP  R | 5’-ATGATGATGATGATGATGAGAACCCCCCATTTTAT  GTGATGATTGATTGATTGATTGTAC |  |
| 0152VCN  F | 5’-GTAGAGTTTTCATTTTCTGGAATGAAAAAACGGAT  CCCCGGGTTAATTAA | pYES-wBm0152 split-Venus |
| 0152VC  R | 5’-ATAACTAATTACATGATGCGGCCCTCTAGGCTACT  TGTACAGCTCGTCCATGCC |  |
| 0152VN  R | 5’-ATAACTAATTACATGATGCGGCCCTCTAGGCTACT  CGATGTTGTGGCGG |  |
| VPS2 -500  F | 5’-CGTAATACGACTCACTATAGGGCGAATTGGGTCAAA  CAGCATTAAAAAGTGATATGC | pRS415-VPS2 split-Venus |
| VPS2 VNC  R | 5’-GATTACGCCAAGCGCGCAATTAACCCTCACCCTAG  CGGATCTGCCGGTAG |  |
| VPS2VN F | 5’-CGACTCACTATAGGGAATATTAAGCTTACCATGAG  TTTGTTTGAGTGGGTATTTGG | pYES-*VPS2* split-Venus |
| VPS2VN R | 5’-GAGACCGAGGAGAGGGTTAGGGATAGGCTTCTACTC  GATGTTGTGGCGGATC |  |
| BM6583 VN F | 5’-CAAGCTAGGCTGGAAAATTTGAGACGTGAACGGAT  CCCCGGGTTAATTA | BM6583 split-Venus |
| BM6583  VN R | 5’- CCTATTATTCATTAAATATACTCAGAGCGCCTACT  CGATGTTGTGGCGGA |  |
| pr29 | 5’-CACATACGATTTAGGTGACAC | HPH conversion |
| pr32 | 5’-AATACGACTCACTATAGGGAG |  |
| **gBlock** |  |  |
| yoBM6583-myc | 5’-cgtaatacgactcactatagggcgaattgggCAGCTC  ATAAAGCATCTTAGTGAAAAGGGTGGTTTTGCGTTA  TTCTTTCCTCTGTTGAAGCTTTTCTATTTTGTTTAAGC  TGAACTAGTGGGAGTTTCCATTAAAAGGCTAAAGTC  TTGTTGAAACTTTTCCGGGTTAGATGATAACTGCGAA  AAGATAGATAAAATCAGCATTAAAGGGACTTCGTCTC  CTCCTAATCAGAAATCATCCTTTCGAATTATGCGTATT  CAGTTGAAGCGTATTTGTGACCAGTGTACTTCAAGGC  TGATTGTTTCACCCTTGGACACAGAACGTTAAACA**ATG**  GACTTTCTGTTCGGGAGGAAGAAAACTCCTGCCGAG  ATGCTGAGACAAAACCAGAGGGCACTAAATAAGGCA  ATGAGAGAGCTTGATAGAGAGCGTTCAAGGCTAGAGA  TGCAAGAGAAAAAGATAATCGCAGATATAAAAAAGATG  GCTAAGATGAACCAGATGGACTCAGTAAAAGTAATGG  CTAAGGACCTTGTTAGGACAAGGCGTCATGTTAAAAAA  TTTATTATGATGAAAGCGAACATCCAGGCAGTCTCTTTG  AGAGTGCAGACTCTAAAATCTCAAGACGCCATGGCCC  AAGCGATGAAAGGTGTGACGAGGGCAATGCAGAATAT  GAATAGACAGCTTAATCTACCCCAGATCCAAAAAATCAT  GATGGAGTTCGAGCGTCAATCCGAGATTATGGATATGAA  GGAGGAAATGATGGGGGAAGCAGTTGATGATGCAATAG  CTGATGAAGGTGATGAGGAAGAGACCGAGACGATAGTC  GCCCAAGTTCTTGACGAACTAGGTATCCAGATGAACGAA  GAATTATCCGCAATCCCCGCAGCTCAGGGTTCTCTAAAA  CCTGCGGACCAGAACAGGCAACCTCAACCCGCTTTATC  AGACGCGGATGCTGATTTGCAAGCTAGGCTGGAAAATTT  GAGACGTGAAGAACAGAAGTTGATTTCCGAAGAAGACCT  C**TGA**GGATCCGCGCTCTGAGTATATTTAATGAATAATAGGT  CTATACTATAATACATCAACTACTGATATCAAAGTAGGCACT  TAGAATCAAAAGGCTTCCGAAAATTTGTTCAGTTTTTCACTT  TACTCAATCTCGTCACTAAAGTTTTCTTTTTTTTTCGAATGAAT  CCTCGAATAAATATGTTCTATATTATATATATACATCTTTTATATA  TCATTAAATGTACAGTAATCGGTCAAATTACAAATGCTTACGG  ATGATTTTTTCACTGATTAAGTACTCATAAATAATGGTAGCACA  TTCTTCAAgtgagggttaattgcgcgcttggcgtaatc |  |

^a^For gBlock sequence, lowercase signifies regions of homology to pRS414, bolded sequence identifies BM6583b start codon, and underlined sequence is the appended BamHI restriction site immediately after stop codon (also bolded).
