# Supplementary material for: Wbm0152, an outer membrane lipoprotein of the Wolbachia endosymbiont of Brugia malayi, inhibits yeast ESCRT complex activity
Source: PLoS Pathog. 2025 Dec 11;21(12):e1013383. doi: 10.1371/journal.ppat.1013383 (PMC12707686; doi:10.1371/journal.ppat.1013383)
Supplement: Attachment — Submitted filename: Review Feedback.docx [file ppat.1013383.s008.docx]

Here we provide responses to reviewer feedback in a hope to mitigate the concerns raised by reviewers on the manuscript entitled "Wbm0152, an outer membrane lipoprotein from *Wolbachia* endosymbiont of *Brugia malayi* inhibits yeast ESCRT complex activity.” We have included original reviewer comments below (starting with >) and prefacing our response with ‘Author Response’. We thank the reviewers for providing their insight into our research.

**Major Shared Revision I: wBm0152 controls**

> Reviewer 1 Comment: “Can the authors modify and quantify the extent of the over-expression? I do have a concern that some of the effects may be a general response of expressing large amounts of a foreign protein. This is especially a concern in the localization studies. Is the localization the same at different levels of expression? As a control, the authors may want to consider expressing scrambled version of wBM 0152”

> Reviewer 2 Comment: “ Because this work is necessarily focused on heterologous assays, controls are especially important. The authors are appropriately circumspect about what they can infer/conclude from these assays. That said, on lines 398-402 they attempt to address the fact that Pal proteins are periplasmic-facing lipoproteins so it is unclear how they would interact with host cell proteins. I think an excellent control for the importance of this result would be to express E.coli Pal proteins in yeast and confirm that they do not have the same phenotype. This would strengthen the argument that *Wolbachia* Pal proteins are behaving differently”

> Reviewer 3 Comment: “I’m a bit surprised that the authors used an empty vector as a control. Wouldn’t a better control be an unrelated protein with a similar length to wBm0152? My worry is that in yeast, even an unrelated protein could potentially have the same effect. Please clarify why you don’t think that would be the case”

**Author Response:**

Due to the consistent concern raised by reviewers regarding the use of a proper protein expression control, we repeated our Congo red Efficiency of Plating assays as well as our RapiDeg assays in yeast strains expressing the wBm0152 homolog from *E. coli* (Pal) which we refer to as ‘EcPAL’. We were able to confirm that expression of EcPAL in yeast does not inhibit ESCRT activity via the RapIDeg assay (new Figure 2), nor does it inhibit the growth of yeast on media containing Congo red (new Supplemental Figure S1C); A Western blot analysis showed expression levels of both EcPal and wBm0152 appears to be roughly equal in both strains (New Supplemental Figure S1B). Therefore, we now demonstrate that wBm0152-mediated ESCRT inhibition activity is a specific function of our *Wolbachia* protein and not due to heterologous overexpression of a bacterial PAL protein in a eukaryotic system.

**Major Shared Revision II: Statistical Analysis and Microscopy Quantifications**

> Reviewer 2: Throughout – it would be good to have quantification, especially for the microscopy figures. Additionally, statistical analyses for all figure panels would be good to include. Also, Figure 4 needs better controls – what is weak or strong colocalization? Perhaps quantification would help to assess this. The Pearson correlation coefficient is sensitive to background and differences in intensities between two channels. By having a positive and negative control for these experiments, it makes the colocalization of the effector and subunits more meaningful to the reader. For Figure5, why are there so many fluorescent puncta in the uninduced control? Quantification of the puncta will help strengthen their argument

>Reviewer 2: Line 242-244: “These results show a reduction in the recruitment of Bro1p to assembled ESCRT complexes in the presence of Wbm0152” Can you make that assertion based on colocalization of Bro1 and wBm0152? You would need to measure colocalization of Bro1 with the ESCRT machinery in the presence and absence of the effector

>Reviewer 3: The manuscript also does not provide sufficient details on the statistical analysis of the data. For instance, the criteria used to quantify colocalization or the statistical significance of differences in growth defects and protein degradation assays are not clearly described. Details on the number of replicates and the statistical tests used should be provided to ensure robustness.

**Author’s Response:**

The addition of a positive and a negative control with the corresponding Pearson Correlation Coefficient analysis was added to the manuscript to address reviewer concerns of defining ‘strong’ and ‘weak’ colocalization (New Figure 5A). As a positive control, the colocalization of the known interacting proteins, Bro1p and Snf7p, was analyzed. For the negative control, Bro1p and Mnn9p – a protein known to localize to the Golgi and have no interaction with Bro1p – was utilized. Statistical analyses were then run comparing the mean PCC scores of each wBm0152/ESCRT colocalization condition to the mean PCC score of the negative control (Figure 5B). The comparison between the *w*Bm0152/Bro1 colocalization scores and the negative control showed no statistical difference providing extremely compelling evidence that wBm0152 does not colocalize with Bro1p. The addition of this statistical analysis and additional controls to Figure 5 should help to mitigate concerns from reviewers about the conclusion that wBm0152 disrupts localization of Bro1 with other ESCRT subunits.

The suggestion by reviewer 1 to quantify the puncta present in the uninduced control in figure 5 was addressed by including an explanation for the fluorescence present in the uninduced conditions (see line 319-321). This assay utilized the copper inducible promoter *CUP1*, which is known to be a ‘leaky’ promoter in the absence of induction [Peng, B., Williams, T.C., Henry, M. *et al.* Controlling heterologous gene expression in yeast cell factories on different carbon substrates and across the diauxic shift: a comparison of yeast promoter activities. *Microb Cell Fact* **14**, 91 (2015)]. This led to some background expression of our split-Venus constructs even in the absence of our inducer, copper sulfate. Furthermore, it is not standard to quantify the number of punctae in these BiFC assays, as reconstitution of the Venus chromophore caused by the protein-protein interaction is irreversible, introducing complexity into the analysis of protein interaction dynamics .

For the RapiDeg experiments, statistical tests (One Way ANOVA) were run to show significance between the addition of rapamycin and the movement of Fth1-GFP into the vacuole lumen (Methods). Additionally, percentages of each phenotype observed were updated, as reviewers requested to better connect what is written in the body of the paper (lines 175-182) and the figure (Figure 2). This, along with the additional analysis of the EcPAL results addressed above, provides increased support and robustness for our claims that wBm0152 induces a striking ESCRT inhibition defect when expressed in yeast.

**Reviewer #1 Major Revisions**

> 2. ESCRT has a number of diverse roles in yeast including nuclear envelope reformation and cytokinesis abscission. What is the effect of over-expression on these events?

**Author Response**

This is a very interesting question, and one that we would love to investigate in a future manuscript that focuses on understanding the mechanism by which Wbm0152 inhibits ESCRT. However, we feel as this experiment this falls outside the scope of this current paper as it could distract the reader from the initial discovery that *w*Bm0152 inhibits at least one activity (the ILV formation activity) of ESCRT and may be important for maintaining the *Wolbachia*:nematode relationship.

> 3. It would be helpful to include mitochondria, nuclear membrane images to determine the quality of membrane preservation in the EM images

**Author Response**

We now have enclosed EM images taken from this series of cell profiles that clearly show intact mitochondrial membranes (new Supplemental Fig S2).

> 4. Because wBm0152 is a membrane bound protein, rather that an effector protein free in the host cytoplasm, there is some concern regarding interpretation of the results. The ectopically expressed Wolbachia membrane-associated domains of wBm0152 are available to associate with host organelles unlike the in vivo situation. While expressing only the non-membrane associated is beyond the scope of the manuscript, this caveat should be considered in the discussion.

**Author Response**

We completely agree with this assessment. While Wbm0152 is not predicted to be a membrane protein itself, its N-terminal lipidation after processing in *Wolbachia* would provide the membrane anchor. Interestingly, immunogold-stained electron micrographs of *Brugia* and *Wolbachia* have previously shown that a population of Wbm0152 is found distant from the bacterial membrane and may be embedded within the membrane surrounding the bacterial inclusion (reference number 105), suggesting that this protein may be positioned to interact with host organelles and protein sorting machineries in the physiologically-relevant system. We discuss this finding in the Discussion section (lines 460-466).

**Reviewer #2 Major Revisions**

> Does Congo Red only interact with ESCRT mutants? Are there other cellular processes that might be impacted leading to these results? Along the same lines, is the accumulation of lipid droplets and the deformation of the ER normal for ESCRT mutants? Or is this just a side effect of the effector? Could this be relevant to other things that wBm0152 is doing in the cell?

**Author Response**

Congo Red is thought to bind to β-1,3 glucans within the fungal cell wall, causing disruptions in polysaccharide synthesis and leading to cell wall damage. Genomic screens have identified a large number of genes and pathways that – when disrupted – cause hypersensitivity to Congo red, although the most represented functional group in this study were proteins involved in vesicular trafficking and transport [García, R., Botet, J., Rodríguez-Peña, J.M. *et al.* Genomic profiling of fungal cell wall-interfering compounds: identification of a common gene signature. *BMC Genomics* **16**, 683 (2015)]. The exact molecular mechanism of how Congo red is processed in the cell and why some trafficking mutants are sensitive to the presence of this compound and not others, remains unclear. Therefore, it is hard to conclude that the defect we observe on Congo red upon wBm0152 expression is solely because of its ESCRT inhibition activity. However, our previously published data showing a ‘Class E’ vacuolar phenotype upon wBm0152 expression in yeast, combined with our current RapiDeg data and colocalization of Wbm0152 with ESCRT subunits, strongly indicate that wBm0152’s toxicity on Congo red is a result of ESCRT inhibition.

Additionally, lipid droplet accumulation and ER deformation are phenotypes that are observed in some ESCRT mutants, as ESCRT activity was found to be important for the consumption of lipid droplets. We have included additional references and background on these phenotypes in lines 235-240 of the resubmitted text to better contextualize the phenotypes we are observing with wBm0152 expression compared to ESCRT mutants.

> 5) Line 268-272: “Interestingly, we did not see 269 reconstitution of Venus fluorescence in Vps2-VC/wBm0152-VN strains (Fig. S1). This observation could be due to the C-terminal -VN fusion forcing Vps2 into a constitutively active, ‘open’ conformation to bind Wbm0152, which is known to occur with yeast and mammalian ESCRT-III GFP fusion proteins.” Are we worried that some of the other subunits are in the wrong confirmation then? Is it possible that some of the negative results shown in Fig S1 are a result of being on the wrong terminus? Only one orientation of Vps25, Vps20, Snf7, Bro1, and Doa4 are shown.

**Author Response**

The tag on the C terminus forces the ‘open’ confirmation of all ESCRT-III subunits therefore if wBm0152 interacts the open confirmations than we would expect to see interaction with all these subunits that contain the VN tag, however we only see interaction with Vps2. Furthermore, the VC tag is significantly smaller as it is only the C-terminal alpha helix of the Venus YFP molecule and may not be forcing the fused ESCRT subunits into an open confirmation. Therefore, if wBm0152 interacted with only the closed confirmation of these proteins, we would expect to see reconstitution under these conditions, which we do not. Unfortunately, the N-terminus of ESCRT subunits are known to define the major interactions between ESCRT subunits and the endosomal membrane. Therefore, it is not possible to tag the N termini of ESCRT subunits without significantly impacting complex assembly, which we know to be critical for the inhibitory activity of Wbm0152 on ESCRT (as suggested in Figure 7).

> 6) Figure 6 - It would be helpful to add the complexes that each subunit is in onto the figure to make the order of assembly clearer to readers. Vps2 is the one subunit that you show to physically interact with wBm0152, but you cannot show that it is necessary for wBm0152 activity. Is there some orthogonal approach you can use to show that this interaction is important to the activity?

**Author Response**

Due to the intricate and co-dependent assembly interactions and the precise stoichiometry requirements between the subunits within the ESCRT complex [Arlt, H., Perz, A. and Ungermann, C. (2011), An Overexpression Screen in *Saccharomyces cerevisiae* Identifies Novel Genes that Affect Endocytic Protein Trafficking. Traffic, 12: 1592-1603.], it is difficult to tease apart the relationships between Wbm0152 and its interacting ESCRT subunit without additional substantial experimentation that do not fit within the scope of this manuscript. In addition, the overexpression of ESCRT protein subunits, like Vps2, induce toxicity in yeast on media containing Congo red, making it difficult to simply overproduce Vps2p in a *w*Bm0152-expressing strain to assay its ability to suppress the *w*Bm0152-mediated growth inhibition on Congo red. Therefore, kinetic assembly assays, biochemical pulldowns, and specific ESCRT point mutants will need to be generated or acquired before such conclusions can be determined.

>7) Figure 7. It is nice to show that the effector interacts with a host protein, but it would also be nice to have some sort of in vivo connection since all of this is out of the natural context. They make guesses about how this might be beneficial to the *Wolbachia*, how does the effector’s activity actually affect the bacterium? I appreciate that this system is very difficult to work in, but are there ESCRT complex inhibitors that can be given to nematodes to assess effects on *Wolbachia* titer? Or perhaps some coIP in the natural system? I think this would help solidify the significance of the finding.

**Author Response**

Unfortunately, despite the wide breadth of literature available on the ESCRT complex and its biochemical mechanisms, there are currently no specific pharmacological inhibitors of the complex, making it additionally difficult to assay the effect of ESCRT inhibition on *Wolbachia* populations in the nematode. Additionally, co-immunoprecipitations out of the natural system would likely be unhelpful in illuminating the mechanisms of Wbm0152 interaction, as there is a lack of good ‘positive’ and ‘negative’ controls (in the form of either *Wolbachia-* or *Brugia-*directed biochemical and immunological reagents) to help differentiate background from specific binding with this system. Therefore, we believe our data provides good evidence for a Vps2:Wb0152 interaction in the nematode and provides an opportunity to explore the molecular mechanisms of this interaction in a way the native system cannot.

**Reviewer #3 Major Revisions**

> While yeast serves as a convenient model, it seems that one important experiment missing is validation of these findings in the natural host, Brugia malayi. The absence of direct validation in a nematode limits the conclusions. As the authors mentioned in the Discussion, RNAi on host genes of ESCRT in JW (Drosophila cells) resulted in an increase of insect *Wolbachia*. A similar experiment (RNAi on proteins of the ESCRT complex) on Brugia would help validate the results obtained in the yeast model. The validation would also support the functional essentiality of the ESCRT-III in Brugia and its physiological role in controlling symbiosis. These are pretty substantial experiments so at this point the best would likely be to tamper the message.
For example, line 136-139 states: “These findings not only identify a bacterial protein capable of inhibiting ESCRT activity but also illuminate an intricate relationship between *Wolbachia* and its nematode host to support its intracellular survival.” The correct statement should be that what this demonstrates is that a bacterial protein can inhibit ESCRT activity in yeast. As for illuminating the intricate relationship between *Wolbachia*/filaria, that seems an overstatement.

**Author Response**

Although we feel it is likely that wBm0152 is inhibiting ESCRT in the native host, we understand the reviewer’s request for additional experiments in the nematode. However, these experiments are exceptionally difficult to perform and RNAi-based experiments in *Brugia* have had limited success, therefore we believe these experiments lay outside of the scope of this study. We hope to further explore the relationship between Wbm0152 and host cells by performing such experiments working on the nematode in a future manuscript. For the current paper, we have made the highlighted statement from above more conservative (lines 151-153) to better reflect the conclusions that can be drawn from our current experiments.

> 3. Discussion: The study concludes that wBm0152 interferes with ESCRT to support *Wolbachia* persistence in the host. However, the manuscript does not explore the physiological consequences of this interaction in the context of *Wolbachia*'s lifecycle within Brugia malayi. For example, it is known that *Wolbachia* numbers increase exponentially during worm development when the worm enters a mammalian host. It will be beneficial to discuss how bacteria manage this expansion. Can *Wolbachia* overexpress wBm0152 during the development of the worms? Transcriptomic data of *Wolbachia* in worms at different stages are available, and the data would help support the discussion. It would be beneficial to discuss how the ESCRT-Wbm0152 interaction influences *Wolbachia's* intracellular survival, replication, or transmission in the nematode host.

**Author Response**

We had added additional text in the discussion section (lines 434-438) to connect the results of our study to previously published transcriptomic data on wBm0152, in the context of *Brugia* life stage development.

**Reviewer Minor Revisions**

>Reviewer #1: 1. Lines 158-162- I'm confused because the percentages listed in the text do not match the percentages listed in Figure

>Reviewer #3: Additionally, the listing of the percentages on the fluorescent images is confusing when also referring to the percentage of cells (2%) accumulating Fth1-GFP in the vacuole lumen 2h after treatment with rapamycin (lines 162-163). One percentage refers to the number of cells that express fluorescence (vacuole membrane or lumen), while the other one refers to the remaining wBm0152 cells that still degrade Fth1-GFP after Rapamycin treatment. Since the percentages of ‘no rapamycin’ minus ‘rapamycin’ comes also to 2% in both the vector control and the wBm0152 cells, it becomes confusing.

**Author Response**

We have updated the RapiDeg figure to no longer include the percentages on the images themselves, choosing to just present statistical analysis on the charts provided in Fig 2. Supplemental data contains the raw percentages in table format, if readers would prefer to have the data presented in this manner. We believe that this will clear up confusion on quantification brought by the reviewers.

>Reviewer #1: 2. Typo in abstract: "These mosquito borne pathogens......". The vector for river-blindness is the Black fly not a mosquito

>Reviewer #3: Abstract: Line 19: “These mosquito-borne pathogens…” Onchocerca parasites are transmitted by black flies, not by mosquitoes.

**Author Response**

We thank the reviewers for pointing out this typo in the text, all lines have been edited to state ‘arthropod-borne’ pathogens to accurately describe the vectors for these nematodes.

>Reviewer #1: 3. Lines 337-339 "Through a combination of mutant insect....the importance of host actin dynamics for the uptake and maternal transmission of *Wolbachia*......." I suggest the literature taken as a whole demonstrate that host microtubules play a dominant role in *Wolbachia's* subcellular interactions. This should be considered as there is a growing literature on physical and functional interactions between ESCRT and microtubules

**Author Response**

Additional information about the importance of microtubules in *Wolbachia* transmission was added to the discussion section (lines 380-383) to better encompass the role of the cytoskeleton in *Wolbachia* transmission.

> Reviewer #2: Line 325-326: “we have shown that expression of Wbm0152 in yeast inhibits the eukaryotic ESCRT complex via interactions with the core ESCRT-III protein, Vps2p.” Actually, they show that the effector interacts with Vps2, and they show that the effector inhibits the ESCRT complex, but they don’t actually show that the vps2 interaction is what causes the inhibition. They couldn’t determine that with their Congo Red assay, so they can’t really assert this.

**Author Response**

We have adjusted our conclusions to better represent the data and information that can be demonstrated within the confines of our current experimental results (lines 366-374).

> Reviewer #2: As stated above, a discussion of the lipid accumulations and perturbed ER morphology upon wBm0152 expression would be appreciated. How can this be related to the ESCRT complex? Or is there another cellular process that is affected? Are there any known ER stress responses known to occur in Brugia with Wolbachia (maybe in that RNA seq study that is referenced)?

**Author Response**

We have added additional references and explanation for these results, (lines 236-240) as well as included more potential implications in the discussion section (lines 470-472) that may better connect wBm0152 and ESCRT to these phenotypes.

>Reviewer #3: Authors used yeast deletion strains in this study; could the overexpression of ESCRT-III rescue wBm0152 effects? This could be part of follow-up studies that confirm the competitive aspect of Wbm0152 interactions with ESCRT-III components.

**Author Response**

We plan on conducting follow up experimentation to better understand wBm0152 interaction with ESCRT-III as the reviewer suggests. However, as mentioned in a previous response to reviewer number 2, overexpression of ESCRT subunits can cause altered endolysosomal trafficking and toxicity on Congo red, making the experiments to untangle wBm0152 interaction with ESCRT much more complicated than simple overexpression of the subunits. Therefore, to fully address wBm0152 interaction with ESCRT, additional experimentation that falls outside the scope of the manuscript would be required. However, we aim to perform such experiments in the future.

> Reviewer #3: The authors refer to Wolbachia pipientis – this nomenclature comes from the fact that the first Wolbachia discovered was in the mosquito Culex pipientis. However, the nomenclature of Wolbachia is a bit messy and so, in the Wolbachia field, Wolbachia are generally referred to by the name of their host. For example, Wolbachia of Drosophila melanogaster is wMel; Wolbachia of Brugia malayi (which is very different genomically from Wolbachia of arthropods) is referred to as wBm. I suggest the authors take out “pipientis” when referring to Wolbachia throughout their manuscript.

**Author Response**

We have removed ‘pipientis’ from the nomenclature throughout the paper and referring to the bacterium as ‘*Wolbachia*’ or ‘*w*Bm’ as the reviewer suggested.

> Reviewer #3: The introduction section would benefit from more detailed information about the significance of ESCRT complexes in eukaryotic cells and their known interactions with intracellular pathogens.

> Reviewer #3: At the end of the abstract, I suggest the authors add a statement, like they did in the author summary, on why looking at ESCRT is important.

**Author Response**

We have added additional background about ESCRT in both the introduction paragraph (lines 113-117) and abstract (lines 27-29) to provide readers with a better understanding of the significance of the ESCRT complex from the beginning of the paper.

> The discussion section could be expanded to include a more thorough comparison of the study's findings with existing literature on ESCRT manipulation by other intracellular pathogens.

**Author Response**

We feel as though the present manuscript contains ample information on ESCRT manipulation by intracellular pathogens (lines 395-418). Because we are still attempting to unveil *Wolbachia’s* interaction with ESCRT, it seems a bit premature to compare the molecular mechanisms from other intracellular organisms to Wolbachia directly.

>Reviewer #3: G should be capitalized in Gram-negative throughout the text

> Reviewer #3: Line 98: Should be “led” not “lead”

>Reviewer #3: Line 147-148 “the Rapamycin Dependent Degradation Assay (RapIDeg)” before the authors stated it as “line 128: the rapamycin-induced degradation (RapIDeg) system”.

**Author Response**

All of these grammatical and typing errors have been adjusted in the manuscript. We thank the reviewer for bringing these to our attention.

> Reviewer #3: Line 267: If the snf7p is not colocalized with wBm0152, how do the authors explain their colocalization in the previous result (Fig. 4)

**Author Response**

We address in the manuscript that wBm0152 does colocalize with Snf7p as well as Vps27p and Vps23p. In this study, we show that wBm0152 does not colocalize with Bro1p (Figure 5)—which is surprising because Bro1 is known to bind Snf7. We hope the addition of the statistical analysis will help to clarify this confusion.

>Reviewer #3: Lines 204-205. As ILVs and MVBs are measured in the following Figure, some explanation on the physiological connections between ILV and MVB would help better understand the results. 
**Author Response**

Additional information was added (Lines 127-140, and a new Figure 1) to help better contextualize the relationship between ESCRT, ILVs, and MVBs.

> Reviewer #3: Results starting at Line 302: In that first paragraph, you mention the Brugia ortholog. Showing % homology between Brugia vps2 and yeast vps2 would be helpful.

**Author Response**

We have added a sequence alignment to address this (Figure 8A). We have also included the percent homology in the relevant figure legend.

> Reviewer #3: Line 361: What is the physiological role(s) of ESCRTs in cell biology?

**Author Response**

We feel as though the question of physiological roles of ESCRT are sufficiently addressed throughout the manuscript, hence no additional information was added to this section. However, we hope the additional information added about ESCRT in the introduction and discussion (lines 113-140; 467-485) as well as the additional ESCRT model figure (Figure 1) will help address these concerns.

> Reviewer #3: Fig 1: It would help the reader to add labels to the fluorescent pictures, pointing to the vacuolar membrane and to the vacuole lumen, as described in the results.

**Author Response**

Arrows were added to better draw reader’s attention to the indicated phenotypes presented in Figure 2 and the associated figure legend has been updated accordingly.

>Reviewer #3: The authors mention many proteins in their complexes, and it is at time confusing. A schema of these proteins in ESCRT-III and the potential localization of wBm0152 would be helpful to the reader.

**Author Response**

We have included an ESCRT schematic to help readers better follow the pathway (Figure 1). We did not include wBm0152 localization within this pathway as we are still unsure of the complete molecular interactions wBm0152 has with ESCRT and would not want to misrepresent our findings.
